# The Story of the Insane from Year to Year

**Published:** 1897-12-11

**Authors:** 


### THE STORY OF THE INSANE FROM YEAR TO YEAR.

Tenth Series.

### THE LANCASHIRE ASYLUM AT PRESTWICH.

On January 1st, 1896, this asylum contained 2,515 patients, and on December 31st the number had risen to 2,599. There were 473 first admissions, 87 readmissions, 214 recoveries, 82 discharged relieved, and 176 deaths. The recoveries reach the respectable percentage of 38·21 on the admissions, and the deaths come out low at 6·93 on the average number resident. Domestic trouble, mental anxiety, religion, love, &c., account for 154 of the year's admissions, intemperance in drink for 94, and hereditary tendency for 95. In 97 the cause was unknown. Sixty-six of the deaths were caused by cerebral diseases, 36 to consumption, and 1 to enteritis. Additions were recently made, and we hear that nearly all the vacancies have been filled, "and the question of providing accommodation for the growing demands of the district will again have to be considered." Mr. Ley recommends that increased and improved hospital accommodation should be provided, and no doubt this would be desirable in view of the ever-increasing number of feeble cases sent to our county asylums, but it would be a matter of much regret if any enlargement of the asylum for general purposes should be carried out. The asylum is already at least twice as large as it ought to be. The farm is credited with a favourable balance of £135. The average cost per head per week was 8s. 4½d.

---

### EARLSWOOD ASYLUM FOR IDIOTS, REDHILL.

On January 1st, 1896, there were 579 patients in residence, and on December 31st the number had risen to 583. There were 57 admissions, 7 readmissions, 40 were discharged relieved, and 20 deaths. It is stated that in 22 of the admissions the idiocy was owing to hereditary tendency, in 2 to consanguinity of parents, in 13 to convulsions during teething, and in not fewer than 16 to shock or fright received by the mother during pregnancy. Of the deaths 3 were caused by apoplexy and epilepsy, and 11 to consumption. Dr. Caldecott thinks that it would be desirable to divide so-called idiots and imbeciles into two classes—one trainable and the other untrainable. The first class he would retain in the asylum, but would rename the institution and christen it a training school. The second class, or the untrainables, he would separate from the others and provide for them in homes distinct from the training schools, but not too far removed to prevent their being supervised by the same administrative officials. We believe there is something in Dr. Caldecott's idea, but want of funds must relegate it to the distant future for realisation. It is well known that much work is done by the patients in the Earlswood Asylum, and on looking over Table VI. we find that 18 work in the carpenters' shop, 33 on the farm and garden, 9 in the printers' shop, 18 in the tailors' shop, and about 164 are otherwise employed.

### WEST RIDING ASYLUM, MENSTON.

A large increase in the numbers has to be noticed here. On January 1st, 1896, there were 1,242 patients in residence, and at the end of the year there were 1,475. There were 630 first admissions, 56 readmissions, 166 recoveries, and 181 deaths. Put into percentages these numbers show that the recoveries stand at 24·19 on the admissions, and the deaths at 12·88 on the average number resident; the former of these being below the county asylum average and the latter above it; but excluding transfers the recovery rate rises to 36·48, a fair average. Of the deaths 71 were from cerebral and spinal diseases, and of that number not fewer than 55 were from general paralysis. Consumption was the cause of death in 10 cases, and enteritis in 3. Various moral causes, such as domestic trouble, religion, and business anxieties account for the insanity in 145 of the year's admissions, intemperance in drink for 85, and hereditary tendency for 105. Dr. McDowall and his colleagues continue to give instructions to the attendants and nurses. Eleven of them entered for the Medico-Psychological Certificate, and all passed the examination. It is proposed to light the asylum throughout with the electric light, and manifest advantages would follow if this proposal be carried out. Although the asylum is only ten years old the system of warming seems an extremely cumbrous and expensive one, and it is suggested to improve it by centralising the boilers used for this purpose. These hot-water and hot-air contrivances are hardly satisfactory, but they are sometimes the least of two evils and have to be tolerated. Farming would seem to be a profitable occupation in the West Riding. The asylum farm accounts show a profit of £776, and rent of land, paid labour, and patients' labour are estimated. The prices of provisions supplied are given, and they are not put too high. The visitors "record their appreciation of the constant care shown by the medical superintendent and his assistants." The weekly cost per head is 8s. 10⅝d.

### LINCOLNSHIRE COUNTY ASYLUM AT BRACEBRIDGE.

There were 734 patients in this asylum on January 1st, 1896, and 757 on the last day of December. The admissions were 175, the readmissions 62, the recoveries 84, and the deaths 87. The recoveries reached the high percentage of 42·6 on the admissions, but the readmissions were also high. The deaths were 11·7 on the average number resident, which is also above the average. Of the total number of deaths 36 were caused by cerebral and spinal diseases, 9 by consumption, and 4 by pneumonia. Of the year's admissions, 40 owed their insanity to moral causes, 26 to intemperance in drink, and 99 to hereditary influence. Although 69 patients are boarded out at other asylums, there are at present 20 more patients on the female side than it was designed to accommodate, and as room for women is not always easy to get, the Visitors may find the solution of the problem a rather difficult one. Dr. Ditton is conducting classes preparatory for the examination of the Medico-Psychological Association, and it is hoped that many nurses and attendants will obtain the certificate. As Dr. Torney says, attendants and nurses take more interest in their work when properly trained, and we may add they do their work better. Dr. Marsh, the former medical superintendent, died early in the year, and Dr. Torney was elected in his place. One of the assistant medical officers is a lady doctor, and Dr. Torney speaks highly of her services. The weekly rate of maintenance is 9s. 0½d. per head.

